# A large cryptosporidiosis outbreak associated with an animal contact event in England: a retrospective cohort study, 2023

**DOI:** 10.1017/S0950268824000591

**Published:** 2024-05-27

**Authors:** Lewis Peake, Megan Bardsley, Samantha Bartram, Shireen Bharuchi, Josh Howkins, Guy Robinson, André Charlett, Rachel Chalmers, Sarah Bird, Nick Young

**Affiliations:** 1 Health Protection Operations, United Kingdom Health Security Agency, Bristol, UK; 2 Food Safety and Health & Safety, South Hams District Council and West Devon Borough Council, UK; 3 *Cryptosporidium* Reference Unit, Public Health Wales, Swansea, UK; 4 Statistics, Modelling and Economics, United Kingdom Health Security Agency, London, UK

**Keywords:** *cryptosporidium*, zoonoses, cryptosporidiosis, United Kingdom

## Abstract

Development of gastrointestinal illness after animal contact at petting farms is well described, as are factors such as handwashing and facility design that may modify transmission risk. However, further field evidence on other behaviours and interventions in the context of *Cryptosporidium* outbreaks linked to animal contact events is needed. Here, we describe a large outbreak of *Cryptosporidium parvum* (*C. parvum*) associated with a multi-day lamb petting event in the south-west of England in 2023 and present findings from a cohort study undertaken to investigate factors associated with illness. Detailed exposure questionnaires were distributed to email addresses of 647 single or multiple ticket bookings, and 157 complete responses were received. The outbreak investigation identified 23 laboratory-confirmed primary *C. parvum* cases. Separately, the cohort study identified 83 cases of cryptosporidiosis-like illness. Associations between illness and entering a lamb petting pen (compared to observing from outside the pen; odds ratio (OR) = 2.28, 95 per cent confidence interval (95% CI) 1.17 to 4.53) and self-reported awareness of diarrhoeal and vomiting disease transmission risk on farm sites at the time of visit (OR = 0.40, 95% CI 0.19 to 0.84) were observed. In a multivariable model adjusted for household clustering, awareness of disease transmission risk remained a significant protective factor (adjusted OR (aOR) = 0.07, 95% CI 0.01 to 0.78). The study demonstrates the likely under-ascertainment of cryptosporidiosis through laboratory surveillance and provides evidence of the impact that public health messaging could have.

## Introduction

The protozoan parasite *Cryptosporidium* is known to cause gastrointestinal illness (cryptosporidiosis) in humans, predominately in the United Kingdom by *Cryptosporidium hominis* and *Cryptosporidium parvum* (*C. parvum*) species, with *C. parvum* found in young livestock. Over 4,000 laboratory-confirmed human infections are recorded in England every year [1] and can lead to long-term health effects [2] [3]. Outbreaks have been associated with private and public water supplies and swimming pools [4], as well as food sources [5, 6]; zoonotic outbreaks have been linked to people bottle-feeding lambs, contact with pre-weaned calves, and poor hygiene in farm environments [7]. An industry ‘Code of Practice’ exists in England to support the minimization of infection risks resulting from animal contact at visitor attractions [8] and reflects learning from high-profile disease outbreaks [9].

In International Organization for Standardization week 17 of 2023, routine surveillance using an exceedance threshold derived from the Farrington Flexible algorithm [10] by the UK Health Security Agency (UKHSA) identified significantly higher *Cryptosporidium* laboratory notifications in the south-west of England compared to seasonally expected levels. A review of routine surveillance questionnaires found that a high proportion of these cases visited a single venue in the preceding Easter holiday period, for a lamb petting experience. A multidisciplinary outbreak control team (OCT) was convened to assess the risk to public health and ensure timely investigation to inform public health action. Furthermore, a cohort study was performed after the incident with the aim of investigating exposures and behavioural risk factors associated with illness.

The primary hypothesis of the analytical study was that entering a lamb pen during the visit was associated with cryptosporidiosis. Secondary hypotheses were that participation in other on-site activities (such as use of a sandpit for children or interaction with other animals), infrequent or absent handwashing, and lack of awareness of diarrhoeal and vomiting disease transmission risk on farms were associated with illness. Here, we describe the findings from the initial outbreak investigation and subsequent analytical study.

## Methods

### Event context

The exposure event under investigation was a pre-booked lamb petting experience. Access to the venue allowed entry (primarily for children) to one of four lamb pens for petting and bottle feeding, whilst adults observed from outside the pen fences. The wider premises also included a separate barn containing a small number of other penned animals (such as goats and sheep not intended for petting), as well as a picnic area, bouncy castle, and children’s sandpit and ball pool. The barn was approximately 20 m from the lamb petting activity; hand hygiene stations were available at the event, positioned outside the activity barn.

### Outbreak investigation

After the detection of the outbreak through both routine surveillance and intelligence from the local authority, case definitions for the initial outbreak investigation were agreed (as summarized in Table 1). Case finding proceeded through a review of all regional *Cryptosporidium* routine surveillance questionnaires to identify whether a visit to the venue was reported in the 12 days prior to illness onset.

**Table 1.** Primary and secondary case definitions used in the initial outbreak investigation and in the cohort study

Outbreak investigation	
Primary case	***Confirmed*** Any person who: visited the lamb petting experience between day 1 and day 16 and reports diarrhoea (‘3 loose poos in 24 h’) or vomiting or abdominal cramping or blood in stools starting <12 days after their most recent visit and provided a faecal sample which tested positive for *Cryptosporidium* ***Probable*** Any person who: visited the lamb petting experience between day 1 and day 16 and reports diarrhoea (‘3 loose poos in 24 h’) or vomiting or abdominal cramping or blood in stools starting <12 days after their most recent visit
Secondary case	***Probable*** Any person who: provided a faecal sample positive for *Cryptosporidium* with a sample data after event day 1 and sample subtyping was in keeping with the outbreak subtype (gp60 subtype IIaA13G1R2 and MLVA profile 5–13–3–13–18–9–27). and more than 12 days between the onset of symptoms and a site visit or no exposure to the site
**Cohort study**	
Primary case	***Confirmed*** Any person who: visited the lamb petting experience between day 1 and day 16 and reports diarrhoea (‘3 loose poos in 24 h’) with onset no later than 12 days after their most recent visit and self–reported that they provided a faecal sample which they were told by a medical professional was positive for *Cryptosporidium* ***Probable*** Any person who: visited the lamb petting experience between day 1 and day 16 and reports diarrhoea (‘3 loose poos in 24 h’) with onset no later than 12 days after their most recent visit
Secondary case	***Confirmed*** Any person who: visited the lamb petting experience between day 1 and day 16 and reports diarrhoea (‘3 loose poos in 24 h’) with onset **more than** 12 days after their most recent visit and lives in the same household as a primary case and self–reported that they provided a faecal sample which they were told by a medical professional was positive for Cryptosporidium ***Probable*** Any person who: visited the lamb petting experience between day 1 and day 16 and reports diarrhoea (‘3 loose poos in 24 h’) with onset **more than** 12 days after their most recent visit and lives in the same household as a primary case
Non–case	Any person who: visited the lamb petting experience between day 1 and day 16 and did not report diarrhoea (‘3 loose poos in 24 h’) with onset no later than 12 days after their most recent visit

Environmental investigations were led by the local authority, which included a site visit with a review of infection prevention and control practices. Because the event had ended by the time of the site review, a decision was made not to pursue animal or environmental sampling given the likely low yield from testing, as well as the absence of ongoing public risk. Animals were returned to the wider herd after the event, and no concerns about the health of any animal were identified by the site operators during or after the event (although none of them underwent a screening veterinary review).

### Microbiology

Cases were diagnosed locally by polymerase chain reaction (PCR) or enzyme immunoassay. *Cryptosporidium*-positive stools were referred to the national *Cryptosporidium* Reference Unit for species identification by real-time PCR [11] and subtyping by sequencing real-time PCR amplicons of the gp60 gene [12] and by multi-locus variable number of tandem repeats analysis (MLVA) [13, 14].

Through these approaches, a common (and unique) subtype attributable to this outbreak was described and used to identify other associated cases which had the same genetic profile, but for whom exposure information was missing.

### Analytical study

The study population was defined as any member of the public who registered for, and subsequently attended, the lamb petting experience between day 1 and the final day (day 16); these were assumed to be mostly local residents, with the potential for national visitors. An online questionnaire was sent to the email list of ticket purchasers held by the venue (provided in supplementary material).

The survey gathered information on the date(s) of the attraction visit(s); preceding or subsequent illness; self-reported results from any faecal sampling; and exposures and behaviours whilst at the setting including entry into the lamb petting pens, engagement in other activities such as use of the children’s sandpit, interaction with other animals, and drink or food consumption on-site. Data were collected anonymously, thereby preventing linkage to laboratory data and necessitating different case definitions for the analytical study (see Table 1).

Responses from the same household were linked through a question requesting individuals list two random words consistently for all household members. The survey also asked whether, at the time of their visit to the attraction, responders had awareness of the risk of pathogen spread from animal contact leading to diarrhoeal and vomiting disease. Answers from adults in a household were extrapolated to children to assess the impact of household awareness on outcomes.

Following descriptive analysis, odds ratios (ORs) and corresponding 95% confidence intervals (CIs) were calculated through single variable logistic regression to examine the association between exposures during the visit and the development of illness for primary cases. Although the study was of a retrospective cohort design, ORs rather than risk ratios were used as the measure of association to protect against the expected differential response rates in those with and without symptoms.

A multivariable logistic regression model was constructed with primary cases, performed in a backward step-wise approach; all variables that had a univariate association with an OR > 2 and a p-value <0.2 were included in the model. Variables were then removed one at a time in decreasing order of p-value and retained if significant at *p* ≤ 0.05 (likelihood ratio test) or if their presence in the model changed a regression coefficient by more than 20%. The age group was retained in all multivariable models as a confounder a priori. To account for clustering among households that attended, mixed-effects logistic regression models were fitted, and exposure variables were retained if they led to an improved model fit.

Given that the incubation period of cryptosporidiosis can be up to 12 days, but has a median of 7 days [15], to assess the impact of potential misclassification of secondary cases, a sensitivity analysis was planned; this analysis would reassign primary cases as secondary cases where symptom onset was more than seven days after symptom onset of the first case in their household (even if the ‘secondary’ case had visited the attraction within 12 days).

This study was reviewed and approved by the UKHSA Research Ethics and Governance Group.

## Results

### Outbreak investigation

Across the 16-day period, 1,372 tickets were pre-ordered for the animal contact event; public health advice (‘warn and inform’ information) was sent to all ticket bookers after the declaration of the outbreak.

**Figure 1.**
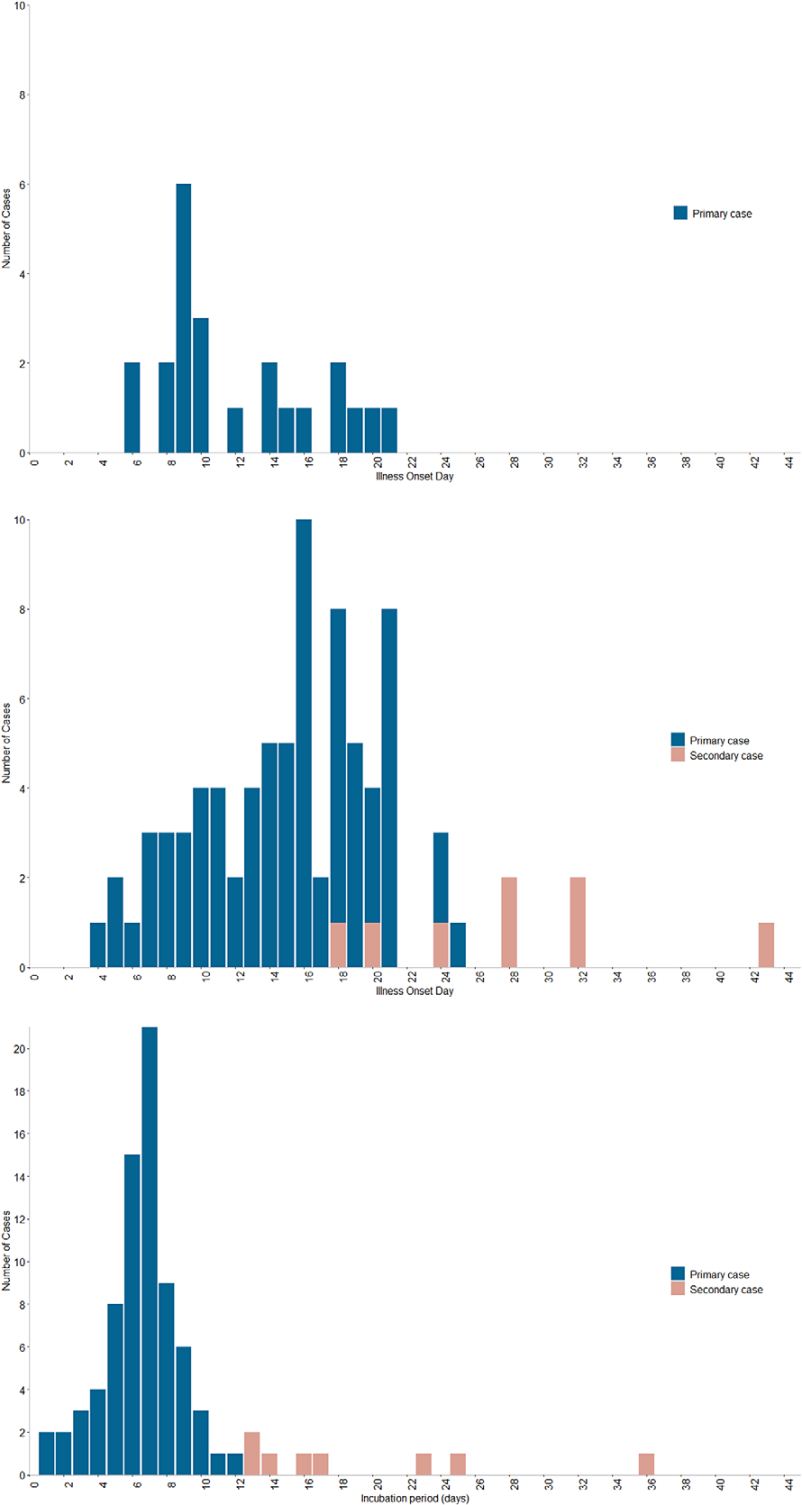
Epidemic curves for the outbreak investigation and cohort study. Top panel: confirmed primary case numbers within the outbreak investigation by day of illness onset (n = 23), where days 1 to 16 are the days the attraction was open. Middle panel: confirmed and probable primary and secondary cases within the cohort study by day of illness onset (n = 83), where days 1 to 16 are the days the attraction was open. Bottom panel: confirmed and probable primary and secondary cases within the cohort study by incubation period (date of illness onset minus date of last or only visit to the setting, n = 83).

Cross-referencing of laboratory reporting and routinely completed cryptosporidiosis questionnaires identified 23 confirmed primary cases of *Cryptosporidium* associated with event attendance (Figure 1); 16 of these confirmed specimens were identified as *C. parvum* (with the remaining unable to be speciated) all of which had a common genetic profile (gp60 subtype IIaA13G1R2 and MLVA profile 5–13–3-13-18-9-27). Five (22%) of the 23 confirmed primary cases reported hospital admission, with a further two cases being assessed and discharged by emergency care. The median age of primary cases was 11 years (range 2 to 49 years); 65% (15/23) were female; and the median time from event attendance to symptom onset was 7 days (range 2 to 8 days).

The gp60 subtype and MLVA profile common to the outbreak were identified in samples from diagnostic laboratories in Devon and Cornwall for a further 17 individuals, all with samples dated between 6 and 26 days after event closure. Information about exposure to the event was only available for two of these cases, both of which denied attendance.

A site visit reported that lamb petting was conducted in the same pens in which the animals were housed for the event duration. Other animals in the activity area not intended to be petted were kept in enclosures close enough that they could be touched by visitors and located within the same large open barn as the bouncy castle, sandpit, and ball pool. Handwashing facilities with good signage were available, but not located close to the animal contact areas.

### Analytical study

For the retrospective cohort study, the survey was deployed via the venue to all email addresses (n = 647) associated with ticket bookings, which generated 199 anonymous responses (including from parents or guardians on behalf of children). In total, 35 responses were excluded for non-completion of important data fields (such as key exposures), and a further three were excluded for having reported household illness prior to visiting the event. Finally, four responses were removed for inconsistent reporting of symptoms.

The remaining 157 responses were included in the final analysis: 75 primary cases (nine confirmed and 66 probable), eight secondary cases (all probable), and 74 non-cases (as per the definitions in Table 1). The earliest primary case reported symptom onset one day after event attendance (median incubation 7 days, range 1 to 12 days; Figure 1). All secondary cases reported a symptom onset within 36 days of their venue attendance. There was no discernible pattern between the specific day of visit and the development of disease; each of the 16 days of operation was associated with at least one case.

The characteristics of cases and non-cases are described in Table 2. Among primary cases, 40 (53.3%) were children under 18 years of age, a higher proportion than non-cases (n = 28, 37.8%). Self-reported symptoms in addition to diarrhoea were consistent with *Cryptosporidium* infection. Over half of cases (n = 49, 59.0%) reported symptoms lasting for 6 days or more, and four (4.8%) reported hospital admission.

**Table 2.** Characteristics of cohort study survey responders, by case category

	Primary case		Secondary case		Non-case	
	n = 75	%	n = 8	%	n = 74	%
**Age group**						
0–4	15	20.0	2	25.0	8	10.8
5–10	19	25.3	1	12.5	20	27.0
11–17	6	8.0	0	–	–	–
18–29	1	1.3	0	–	4	5.4
30–50	32	42.7	4	50.0	32	43.2
51–69	2	2.7	1	12.5	6	8.1
>70	0	–	0	–	4	5.4
**Gender**						
Male	37	49.3	2	25.0	27	36.5
Female	38	50.7	6	75.0	47	63.5
**Illness onset**						
1 to 7 days after the most recent visit	55	73.3	0	0.0		
8 to 12 days after the most recent visit	20	26.7	0	0.0		
13+ days after the most recent visit	0	0.0	8	100.0		
**Has a stool sample confirmed *Cryptosporidium* spp.?**						
Yes	9	12.0	0	0.0		
**Non–diarrhoeal symptoms**						
Vomiting	46	31.1	1	10.0		
Fever	37	25.0	2	20.0		
Stomach pain	65	43.9	7	70.0		
**Length of illness**						
<2 days	1	1.3	1	12.5		
2 to 5 days	27	36.0	5	62.5		
6 to 10 days	31	41.3	1	12.5		
>10 days	16	21.3	1	12.5		
**Hospital admission**						
Yes	4	5.3	0	0.0		
**Diarrhoeal illness in the household after symptom onset in case**						
Yes – 2 people	1	1.3				
Yes – 1 person	4	5.3				
No	70	93.3				

Single variable associations between exposures of interest and cases are described in Table 3. There was evidence that cases were more likely to have entered a lamb petting pen, rather than observed from the outside (OR = 2.28, 95% CI 1.17 to 4.53). Of those who did enter a pen, sitting on the floor/straw was associated with increased illness risk (OR = 2.78, 95% CI 1.11 to 7.17).

**Table 3.** Single variable associations between exposures and primary case status

	All responders (n = 149)						
	Primary case		Non-case		OR	95% CI	p-value
	n = 75	%	n = 74	%			
Age group (years)							Overall 0.12
0 to 4	15	20.0	8	10.8	–	–	
5 to 17	25	33.3	20	27.0	0.67	0.23 to 1.86	
18+	35	46.6	46	62.2	0.41	0.15 to 1.04	
Entered lamb petting pen							
Yes	53	70.6	38	51.4	2.28	1.17 to 4.53	**0.016**
Other animals (any contact)^a^							
Sheep	16/71	22.5	13/68	19.1	1.23	0.54 to 2.84	0.6
Ponies	23/73	30.6	13/70	18.6	2.02	0.94 to 4.49	0.078
Goats	22/74	29.7	20/70	28.6	1.06	0.51 to 2.18	0.9
Other activities							
Bouncy castle	31	41.3	27	36.5	1.23	0.63 to 2.38	0.5
Ball pool	30	40.0	29	39.2	1.03	0.54 to 2.00	>0.9
Go–karting	34	45.3	27	36.5	1.44	0.81 to 3.00	0.3
Sand pit	23	30.6	11	14.9	2.53	1.15 to 5.86	**0.024**
Drink consumption							
Drink not from the site	28	37.3	31	41.9	0.83	0.43 to 1.59	0.6
Water from the site	14	18.6	9	12.2	1.66	0.68 to 4.24	0.3
Other drinks from the site (e.g. hot drinks)	33	44.0	43	58.1	0.57	0.29 to 1.08	0.086
Food consumption							
Food not from the site	11	14.6	14	18.9	0.74	0.30 to 1.74	0.4
Food from the site	59	78.6	54	73.0	1.37	0.64 to 2.93	0.5
Did not eat	9	12.0	9	12.2	0.98	0.36 to 2.67	>0.9
Habitual behaviours^a^							
Thumbsucking	6/74	8.1	4/69	5.8	1.43	0.38 to 5.31	0.6
Nail biting	6/74	8.1	5/69	7.2	1.12	0.33 to 3.88	0.8
Hand hygiene^b^							Overall **0.033**
Never	1	1.3	2	2.7	–	–	
Only used hand sanitizer	1	1.3	8	10.8	0.25	0.01 to 8.20	
Soap/water at any time	51	68.0	47	63.5	2.17	0.20 to 47.6	
Soap/water and hand sanitizer at any time	22	29.3	15	20.3	2.93	0.26 to 66.5	
Awareness of disease transmission risk on farm sites at the time of visit^a^ ^,^^c^							
Yes	15/70	21.4	29/72	40.3	0.40	0.19 to 0.84	**0.015**
	Entered lamb petting pen (n = 91)						
	Primary case		Non–case		OR	95% CI	p–value
	n = 53	%	n = 38	%			
Level of contact with lambs							
Touched	51	96.2	38	100.0	–	–	
Licked/hand–fed	31	58.5	19	50.0	1.41	0.61 to 3.28	0.4
Held or cuddled	32	60.4	16	42.1	2.10	0.90 to 4.96	0.087
Bottle fed	44	83.0	32	84.2	0.92	0.28 to 2.80	0.9
Kissed	2	3.8	3	7.9	0.46	0.06 to 2.90	0.4
No contact	1	1.9	–				
Behaviour in lamb pen							
Sat on floor/straw	42	79.2	22	57.9	2.78	1.11 to 7.17	**0.030**
Played with straw	17	32.1	9	23.7	1.52	0.60 to 4.04	0.4
Carried in a toy/comforter	–		–				
Adults only (n = 81)							
	Primary case		Non–case		OR	95% CI	p–value
	n = 35	%	n = 46	%			
Awareness of disease transmission risk on farm sites at the time of visit							
Yes	6	7.4	21	45.7	0.25	0.08 to 0.67	**0.009**

a Excluding ‘not sure’ responses.

b Participants were asked about whether they cleaned their hands, and using what method, at various times during their visit (e.g. on arrival, before contact with animals, after contact with animals). These data have been summarized here.

c Adult responses extrapolated to children in the same household.

There was some evidence that the use of the sandpit (OR = 2.53, 95% CI 1.15 to 5.86) was associated with an increased risk of illness. Awareness of diarrhoeal and vomiting disease transmission risk on farm sites was negatively associated with illness (OR = 0.40, 95% CI 0.19 to 0.84).

In a multivariable model including all study participants (model A), there was evidence that awareness of diarrhoeal and vomiting disease transmission risk on farm sites at the time of visit was protective against illness (adjusted OR (aOR) = 0.07, 95% CI 0.01 and 0.78), whilst entering a lamb petting pen was a predictor of illness (aOR = 4.49, 95% CI 0.93 to 21.60). Given the near ubiquity of exposure to lamb petting pens among children, a separate multivariable model was also produced for adults only (Table 4 – model B), which demonstrated findings consistent with model A.

**Table 4.** Multivariable associations between exposures and primary case status

Model A All responders, adjusted for household clustering				
	OR	Conf Low	Conf High	P-value
Awareness of disease transmission risk on farm sites at the time of visit	0.07	0.01	0.78	0.030
Entered a lamb petting pen	4.49	0.93	21.60	0.061
Age group^a^ 5–17 years	0.50	0.08	2.98	0.448
Age group^a^ 18+ years	0.78	0.12	4.86	0.787
Model B Adults only				
	OR	Conf Low	Conf High	P–value
Awareness of disease transmission risk on farm sites at the time of visit	0.25	0.08	0.71	0.01
Entered a lamb petting pen	2.27	0.85	6.30	0.10

a Compared to 0–4 years as the reference group.

The planned sensitivity analysis led to no re-classification of case definitions; that is, there were no cases who had developed symptoms more than seven days after a first case in their household.

## Discussion

This investigation describes a significant exposure event that resulted in at least 23 laboratory-confirmed primary cases of *Cryptosporidium* (five of which were hospitalized), with 83 self-reporting cases identified through the cohort study. Analytical study findings support the primary hypothesis that exposure to lambs within designated petting pens was the source of *Cryptosporidium* at the venue, although the absence of any environmental samples limits the certainty of this conclusion. Awareness of the potential for disease transmission on farm sites reduced a person’s risk of illness.

The outbreak we report here is one of the largest reported in England in recent years; data for England and Wales have separately identified 23 such outbreaks between 1992 and 2009 [16] and 74 between 2009 and 2017 (with a median of five lab-confirmed cases, range 3 to 41, linked to each outbreak) [17].This impact and observations from the site inspection highlight the important role event organizers play in mitigating the risk of disease transmission and maintaining public health for their patrons.

Despite the known risk of cryptosporidiosis after animal contact at petting farms, there is less evidence on the individual factors that modify risk at such attractions. In one large study, [7] eating without washing your hands, and a lack of information on arrival, greatly increased the chance of illness; our investigation has reaffirmed the importance of public health information, but did not prove a benefit from certain handwashing practices in multivariable analysis (likely due to difficulty in capturing precise data on handwashing that may have occurred at multiple points across an event visit). Handling animals, and habits such as nail biting or thumbsucking, has also been previously suggested to increase the risk of transmission [16, 18]; our investigation found no association between nail biting or thumbsucking and disease, but individuals who ‘held or cuddled’ a lamb within a pen were more likely to develop cryptosporidiosis-like illness. There was also some evidence that the use of the children’s sandpit was associated with an increased risk of illness, possibly because of exposure to faecal matter on children’s shoes and sand being a difficult material to disinfect. Future research may benefit from mixed-methods approaches that evaluate interventions as recommended in industry practice [8] and, through direct observation, assess the resulting impact on human behaviours.

A site visit following the event highlighted findings that could have contributed to the spread of infection from animals to humans. The housing of lambs within the barns used for petting would has increased the risk of human contact with faecal material, and contact with other animals at the event was possible even though they were not intended to be petted. Although handwashing facilities and relevant signage were present, the location of these was away from the sites of animal contact, thereby potentially reducing their use and effectiveness. Site operators should focus on structural factors, based on pre-event risk assessment and available guidance, to reduce the potential for the spread of disease.

Of note, through this study we have been able to demonstrate both under-ascertainment of cryptosporidiosis-like illness and significant duration of illness in the context of an outbreak. Standard approaches to case ascertainment during the outbreak investigation identified 23 primary *Cryptosporidium* cases, compared to the 83 individuals meeting our definition of cryptosporidiosis within the cohort study. More than 60% of these reported a symptom duration of six days or more.

In this investigation, the identification of a unique MLVA genetic profile within a spatial and temporal cluster provided reassurance that the observed regional exceedance was due to a common exposure and provided some evidence of possible secondary or tertiary transmission within the community (i.e. two cases with a matching MLVA profile but no direct exposure to the setting, 15 cases with a matching profile but no exposure information, and cases with symptom onset up to 26 days after closure of the event). Whilst microbiological testing of specimens from implicated animals could have provided further evidence of the common exposure, such sampling was not considered to be of use in this outbreak given the time elapsed after the event.

The nature of the study design presented biases and limitations. As questionnaires were anonymous (potentially of benefit in minimizing the risks of social desirability bias), deduplication of responses could not be fully assured (although incomplete responses were removed from analysis), or reports of illness were validated against laboratory findings. Additionally, the lag time from outbreak detection to questionnaire deployment meant that responses were received between six and eight weeks after exposure, increasing the chances of recall bias. Whilst the study found that awareness of risk of illness following animal petting events was protective, this finding could be an artefact of social desirability bias.

Overall, the study highlights the potential size and public health burden of *Cryptosporidium* outbreaks from animal contact visitor attractions; how surveillance and outbreak detection may be impacted by under-ascertainment in the community and primary health care; and the potential protective effect from awareness of disease transmission risk. These findings are despite the existence of established industry best practice guidance [8]. There is likely a need for greater awareness among clinicians on the public health benefit of faecal sampling for patients presenting with diarrhoeal disease following contact with livestock and primarily an improved understanding for the public on both the risks of disease transmission during animal petting activities and the symptoms to act upon post-exposure; event pre-booking provides the opportunity for public health messaging for attendees and necessitates public health officials working with industry partners to support them in providing this information.

## Supporting information

Peake et al. supplementary material Peake et al. supplementary material

## Data Availability

Data are available on reasonable request to the authors. Restrictions may apply to the availability of personal data linked to patient and study participant information.

## Supplementary material

For supplementary material accompanying this paper visit http://doi.org/10.1017/S0950268824000591. click here to view supplementary material
