# Supplementary material for: A large cryptosporidiosis outbreak associated with an animal contact event in England: a retrospective cohort study, 2023
Source: Epidemiol Infect. 2024 May 27;152:e91. doi: 10.1017/S0950268824000591 (PMC11736449; doi:10.1017/S0950268824000591)
Supplement: Peake et al. supplementary material [file S0950268824000591sup001.pdf]

Introduction

1. I have read and understood the **STUDY INFORMATION SHEET***

Please read the sheet, attached to the original email, before commencing the questionnaire

- ◯ Yes
- ◯ No

2. We are seeking individual responses to this questionnaire for **EACH PERSON** that attended [name of venue].

Please complete this questionnaire **MULTIPLE TIMES** if necessary (e.g. for yourself, and on behalf of any children), with a separate submission for **EACH PERSON**.

Please confirm you have read and understand the above instruction.*

- ◯ Yes
- ◯ No

3. Are you completing **THIS** questionnaire:*

- ◯ on behalf of someone else
- ◯ for yourself

Personal details

5. On what date(s) and time(s) did the person visit [name of venue]?

If the person visited the farm on more than one of the above dates, for the purposes of this questionnaire please treat ALL visits as ONE visit. For example, if the person played on the bouncy castle on one visit but did not on another visit, respond yes to the question about using the bouncy castle

6. Please state the **age** of the person at the time they visited [name of venue].*

- ◯ 0-4 years old
- ◯ 5-10 years old
- ◯ 11-17 years old
- ◯ 18-29 years old
- ◯ 30-50 years old
- ◯ 51-69 years old
- ◯ 70+ years old

7. Please select the **gender** of the person

*

- ◯ Male
- ◯ Female
- ◯ Non-binary
- ◯ Prefer not to specify

8. Did the person develop diarrhoea (3 loose poos in 24 hours) which began in the 7 days BEFORE, or on the day of, their first/only visit to the farm?*

- ◯ Yes
- ◯ No

9. Did the person develop diarrhoea (3 loose poos in 24 hours) AFTER any visit to [name of venue]?*

- ◯ Yes – had diarrhoea which began between 1 and 14 days AFTER their visit
- ◯ Yes – had diarrhoea which began 15 days or more AFTER their visit
- ◯ No

Your symptoms

11. What date did the person start to feel unwell?* dd/mm/yyyy

12. Has the person submitted a stool (poo) sample **AND** been informed by their GP or other medical professional that they have had "Crypto" / Cryptosporidiosis?*

- ◯ Yes
- ◯ No

13. Did the person experience any of the following symptoms **AFTER** any visit to [name of venue]?

Please select all that apply

	Yes	No
Diarrhoea (3 or more loose poos in 24 hours)	◯	◯
Vomiting	◯	◯
Fever	◯	◯
Stomach (abdominal) pain or cramping	◯	◯
Other	◯	◯

14. Please list any **other** symptoms

15. How long did the person feel unwell for?

- ◯ Less than 2 days
- ◯ Between 2 days and 5 days
- ◯ Between 5 days and 10 days
- ◯ More than 10 days

16. Was the person admitted to hospital for this illness?*

- ◯ Yes
- ◯ No

17. If yes, how many nights were spent in hospital?

-- None --

18. Did anyone in the person's household have diarrhoea (3 or more loose poos in 24 hours) in the 7 days **BEFORE** their first (or only) visit to [name of venue]?

- ◯ No
- ◯ Yes

19. **At any time after** the person became unwell, did anyone in their household who **DID NOT** visit [name of venue] develop diarrhoea? (3 or more loose poos in 24 hours)*

- No
- Yes - 1 other person
- Yes - 2 other people
- Yes - 3 other people
- Yes - 4 other people
- Yes- other number (please specify)

20. How many of those people submitted a stool (poo) sample **AND** were informed by their GP or other medical professional that they had "Crypto" / Cryptosporidiosis?

- 0 people
- 1 person
- 2 people
- 3 people
- 4 people
- Other number (please specify)

Your visit to [name of venue]

21. Whilst visiting [name of venue], did the person take part in the baby lamb experience held in the small petting shed?*

- Yes - entered the lamb petting pen
- Yes - observed from outside the fence
- No
- Not sure

22. Which pen did the person enter?
If multiple visits, select all that apply

- Pen 1- first on left
- Pen 2- second on left
- Pen 3- third on left
- Pen 4- last pen
- Not sure

23. During the baby lamb experience, what level of contact did the person have with the lambs?*
Please select all that apply

- Touched
- Licked by/hand fed the animal
- Held or cuddled
- Bottle fed
- Kissed
- No contact
- Not sure

24. For those who entered the pen, during the baby lamb experience did the person...*
Please select all that apply. It may be helpful to go back through photographs taken on the day as a memory prompt.

- Sit on the floor/straw
- Play with straw
- Eat or drink
- Carry in a toy/comforter
- None of the above

Your visit to [name of venue] - food and drink

25. Did the person eat any food whilst visiting [name of venue]?

*

Please select all that apply

- No
- Yes, food from home/outside the farm
- Yes, food bought at the farm
- Not sure

26. If the person ate food purchased onsite, please state which items these were

27. When did the person eat?*

- Before the lamb experience in the small petting shed
- After the lamb experience in the small petting shed
- Both before and after the lamb experience in the small petting shed
- Did not attend lamb experience in the small petting shed
- Not sure

28. Did the person drink whilst visiting [name of venue]?

*

Please select all that apply

- No
- Yes, drinks not from the farm site
- Yes, bottled/tap water supplied on the farm site
- Yes, other drink supplied on the farm site e.g. milk, juice
- Not sure

Your visit to [name of venue] - other activities

29. When the person visited [name of venue], did they have contact with any animals **in the big activity barn?**

*

This is separate to the lambs held in the small petting shed. Select all that apply

	Adult sheep	Lambs	Ponies	Goats

Touched	☐	☐	☐	☐
Licked by/hand fed the animal	☐	☐	☐	☐
Held or cuddled	☐	☐	☐	☐
Kissed	☐	☐	☐	☐
No contact	☐	☐	☐	☐
Not sure	☐	☐	☐	☐

30. Did the person take part in any of the below activities whilst on [name of venue]?*

Please select all that apply

- ☐ Bouncy Castle
- ☐ Ball Pool
- ☐ Go
- ☐ Karting
- ☐ Sand pit
- ☐ Games area
- ☐ None of the above
- Not sure

31. Whilst at the farm, was it likely that the person:

Please select all that apply

- ☐ Ate with hands (eg sandwiches, ice cream cone)
- ☐ Sucked their thumb
- ☐ Bit their nails
- ☐ Used a dummy / pacifier
- ☐ Smoked / vaped
- ☐ None of the above
- ☐ Not sure

32. Did the person use (sat in, or push) a pushchair/buggy or wheelchair at any point on site at [name of venue]?

- ○ No
- ○ Yes
- ○ Not sure

Your visit to [name of venue] - hand washing

33. When the person visited the farm, at what points did they wash their hands (or use alcohol-based hand sanitizer)?*

	Don't wash	Didn't know	Hand sanitizer only	Water only	Soap and water	Soap, water and hand sanitizer	Not applicable
Immediately after leaving the small lamb petting shed	○	○	○	○	○	○	○
Immediately after contact with any other animals	○	○	○	○	○	○	○
Immediately before any	○	○	○	○	○	○	○

food consumption

When leaving the farm site ○ ○ ○ ○ ○ ○ ○

34. If the person did wash their hands, at any point did they have to wait in a queue for handwashing for longer than a couple of minutes? (rough estimate)*

- ○ No- didn't have to wait
- ○ Yes- had to wait
- ○
- ○ Not sure

Did not wash hands with water at any point

35. If the person did wash their hands, what did they typically use to dry their hands?

Select all that apply

- ☐ Did not dry hands
- ☐ Paper towels
- ☐ Hand towel
- ☐ Air hand dryer
- ☐ Not sure
- ☐ Other, please specify

36. Did the person use baby wipes/hand wipes at any of the following times?*

Select all that apply

	Yes	No	Don't know	Not applicable
	○	○	○	○
	○	○	○	○
Immediately after leaving the small lamb petting shed	○	○	○	○
Immediately after contact with any other animals	○	○	○	○
Immediately before any food consumption				
When leaving the farm site				

37. Did the person use any boot/shoe wash facilities at any point during their visit?*

Please select all that apply

- [ ] Yes
- [ ] No
- [ ] Not sure

Other possible exposures

38. Where do you think the person may have picked up their illness?

Any other information

39. At the time of your visit to [name of venue], were you (or the parent/guardian if completing for a child) aware of the potential risk of picking up diarrhoea and vomiting bugs from visiting petting farms?

Only adults need to answer this question

- ○ Yes
- ○ No
- ○ Not sure

40. Some people will be repeating this questionnaire multiple times if they are responding on behalf of others. We would like to identify which responses come from people **living in the same household.**

To help us identify your household, please enter two random words in the box below. **Use the same two words** for all questionnaires you complete for members of your household. If you are completing on behalf of someone who IS NOT in your household, use a **different two words.**

This question needs to be completed by everyone- even if you are only submitting one questionnaire.
*

eg, dollar shelf

41. Do you have any further comments?

Please click on 'Submit' to finish the survey
